# Exploratory Single-Cell Transcriptomic Profiling Reveals Dysregulated Glial Populations and Pathways in Focal Cortical Dysplasia Epilepsy

**DOI:** 10.3390/biology14121690

**Published:** 2025-11-27

**Authors:** Chao Jiang, Qingyao Gao, Yan Zhao, Yiming You, Zhuojue Wang, Jian Wang, Guang Yang, Chuang Guo, Zhiqiang Cui

**Affiliations:** 1 Key Laboratory of Bioresource Research and Development of Liaoning Province, College of Life and Health Sciences, Institute of Neuroscience, Northeastern University, No. 195, Chuangxin Road, Hunnan District, Shenyang 110169, China; jiangchao103@163.com (C.J.);; 2 Department of Neurosurgery, Chinese People’s Liberation Army of General Hospital, 28, Fuxing Road, Haidian District, Beijing 100853, China; 3 Institute of Scientific Research, National Health Commission, Beijing 100089, China; 4 School of Biomedical Sciences and Engineering, South China University of Technology, Guangzhou International Campus, Guangzhou 511442, China; 5 Medical School of Chinese People’s Liberation Army, Beijing 100853, China; 6 Senior Department of Pediatrics, Chinese People’s Liberation Army of General Hospital, Beijing 100700, China

**Keywords:** DEGs, epilepsy, IL-17 signaling pathway, single-cell RNA sequencing, focal cortical dysplasia

## Abstract

Background: Focal cortical dysplasia (FCD) is a prevalent cause of drug-resistant epilepsy, but a comprehensive understanding of its pathogenesis at a cellular resolution remains limited. Previous transcriptomic studies, often constrained by bulk tissue analysis, have been unable to dissect the cell-type-specific contributions to epileptogenesis. Methods: We performed scRNA-seq on cortical tissues from one surgical patient with FCD type II and one matched control. Cell clustering, annotation, and identification of differentially expressed genes (DEGs) were conducted using standard Seurat workflow. We focused on the molecular alterations in three major glial cell types: astrocytes, microglia, and oligodendrocytes. To functionally interpret the DEGs, we performed enrichment analyses using Gene Ontology (GO) and the Kyoto Encyclopedia of Genes and Genomes (KEGG). Results: Our profiling revealed a profoundly reconstituted cellular ecosystem in the FCD cortex. We found a marked expansion of microglia (65.57% vs. 47.02%; a ~39% relative increase) and astrocytes (10.98% vs. 4.11%; a ~167% relative increase), alongside a severe depletion of oligodendrocytes (8.12% vs. 30.63%; a ~73% relative decrease). Critically, a core set of 128 differentially expressed genes (DEGs) was shared across these glial populations, featuring consistent upregulation of RAC1 and downregulation of ATP5F1D, pointing to convergent pro-inflammatory and mitochondrial dysfunction pathways. Enrichment analyses further demonstrated a coordinated engagement of neuroinflammatory pathways, most notably IL-17 signaling. Subsequent cell–cell communication inference revealed a broad attenuation of intercellular signaling, with a 35% reduction in interaction numbers, indicating a breakdown of coordinated cellular crosstalk. Conclusions: This exploratory single-cell study provides preliminary evidence of a convergent glial pathology in FCD, characterized by shared molecular disruptions in inflammation and metabolism. Our findings highlight RAC1 and IL-17 signaling as potentially actionable pathways, warranting further investigation into their therapeutic potential for mitigating epileptogenesis in FCD.

## Simple Summary

Focal cortical dysplasia (FCD) is a brain condition that frequently leads to severe, hard-to-treat epilepsy. The exact ways in which brain cells contribute to the development of seizures in FCD are not fully understood. In this study, we used advanced technology to analyze individual brain cells from a patient with FCD. We discovered that the brain cell environment is drastically changed, with an overabundance of specific immune cells and a major loss of other supportive cells. These altered cells share common problems, including increased inflammation and faulty energy production, which disrupts how cells talk to each other. Our exploratory findings shed light on potential mechanisms underlying seizures in FCD and highlight two specific biological pathways that could be explored for future therapeutic strategies.

## 1. Introduction

Malformations of cortical development (MCDs) represent a heterogeneous group of neurological disorders arising from aberrant brain development, with subtypes including focal cortical dysplasia (FCD) and tuberous sclerosis complex (TSC) [1,2]. MCDs are a prevalent cause of developmental delays and epilepsy, with clinical manifestations primarily encompassing seizures, intellectual disability, and motor dysfunction [3,4]. Epilepsy, one of the most common neurological disorders, proves drug-resistant in approximately one-third of patients [5]. FCD stands as the most frequent etiology for surgically remediable epilepsy, with around 87% of FCD patients developing seizures [6,7]. Current treatment modalities, such as antiepileptic drugs, surgical resection, neurostimulation (e.g., VNS, DBS, RNS), gene therapy, and dietary interventions, can alleviate seizures to some degree but often fall short of providing a complete cure [5]. Therefore, elucidating the mechanisms underlying epileptogenesis in FCD is of paramount importance. Transcriptomic analyses have yielded insights into epilepsy. For instance, a study of hippocampal and cortical tissues from patients who succumbed to sudden unexpected death in epilepsy (SUDEP) identified 55 differentially expressed genes compared to controls [8]. Lu et al. [9] conducted a meta-analysis integrating transcriptome-wide and proteome-wide association studies (TWAS and PWAS) to discern genetic and environmental influences on epilepsy and predict novel potential drug targets. Another study employed RNA-Seq to construct a competing endogenous RNA (ceRNA) network associated with neuronal apoptosis in hippocampal sclerosis of temporal lobe epilepsy [10]. However, conventional RNA-Seq provides an averaged gene expression profile across bulk tissue, masking cell-to-cell heterogeneity and obscuring cell-type-specific contributions [11].

This limitation is overcome by single-cell RNA sequencing (scRNA-seq), a transformative technology that resolves transcriptional signatures at the individual cell level. scRNA-seq not only delineates gene expression patterns across distinct cell types within a tissue but also captures biologically relevant variation, enabling the interrogation of cellular states during disease progression and offering unprecedented resolution for mechanistic discovery [12,13,14]. This powerful approach has been extensively applied in oncology to dissect tumor heterogeneity [15] and in cardiovascular research to map cellular landscapes in heart development and disease [16]. In neuroscience, the application of scRNA-seq has begun to illuminate the complex cellular underpinnings of epilepsy. Seminal work by Kumar et al. leveraged scRNA-seq to uncover enhanced pro-inflammatory activity within direct T-cell–microglia immune complexes in human epileptic brain lesions [14]. Wen et al. recently utilized scRNA-seq to investigate and compare cellular heterogeneity between post-traumatic and hereditary epilepsy models [17]. Most recently, research has focused specifically on malformations of cortical development. Galvão et al. [18] applied multimodal single-cell profiling to reveal neuronal vulnerability and pathological cell states in human FCD tissues, while Bizzotto et al. [19] used cell-type-informed genotyping of mosaic focal epilepsies to disentangle cell-autonomous and non-cell-autonomous disease-associated transcriptional programs (Table 1).

Despite these advances, as summarized in Table 1, a systematic and integrated analysis of the glial ecosystem in FCD remains lacking, particularly one that examines convergent pathways across different glial types and their intercellular communication. Our study addresses this critical gap. We performed scRNA-seq on surgically resected cortical tissue from a patient with FCD-related epilepsy and a control patient (who underwent ventriculostomy for a brain tumor resection and had no history of epilepsy or antiepileptic drug use). We aimed to define the cell-type-specific transcriptional alterations and identify the differential gene expression networks that underlie FCD pathophysiology, with a specific focus on the reconstituted glial and vascular compartments. By conducting an unbiased scRNA-seq analysis, we seek to provide a high-resolution cellular atlas of FCD epileptic tissue, thereby uncovering novel mechanisms and potential therapeutic targets.

## 2. Materials and Methods

### 2.1. Patients and Tissue Dissociation

One patient with focal cortical dysplasia (FCD)-related epilepsy and one control patient were included in this study. Both patients provided written informed consent for participation. This study was conducted in accordance with the Declaration of Helsinki and was approved by the Ethics Committee of Chinese PLA General Hospital (Approval No. S2021-586-01). Written informed consent was obtained from all individual participants prior to their enrollment in the study.

Baseline characteristics (age, gender, imaging findings, and pathological information) of both patients were collected and recorded preoperatively. Cortical tissue samples were then obtained from the epilepsy patient with FCD and the control patient with a ventricular space-occupying lesion. The tissues were immediately washed with calcium- and magnesium-free PBS and stored in tissue preservation solution before being sent to CapitalBio Technology Inc. (Beijing, China) for further processing.

### 2.2. Single-Cell RNA Sequencing

Single-cell RNA-seq libraries were constructed using the Single Cell 3′ v3 Library and Gel Bead Kit V3.1 (10× Genomics) according to the manufacturer’s instructions. The libraries were sequenced on an Illumina NovaSeq 6000 platform (Illumina, San Diego, CA, USA) using a paired-end 150 bp (PE150) strategy (performed by CapitalBio Technology, Beijing, China), yielding a mean of 75,981 reads per cell pre-normalization and 69,102 reads per cell post-normalization. Following data preprocessing, the sequencing results were subjected to cell–cell communication analysis. We performed scRNA-seq on whole cell suspensions prepared from fresh tissue. It is important to note that this approach, while providing high-quality transcriptomic data from intact cells, typically leads to the loss of neuronal cell bodies during the tissue dissociation process. This explains the absence of neuronal populations in our final dataset and focuses our subsequent analysis on the glial and vascular compartments that remain viable.

### 2.3. Cell Capture and cDNA Synthesis

Cell suspensions (300–600 living cells per microliter, as determined by Count Star) were loaded onto the Chromium Single Cell Controller (10× Genomics, Pleasanton, CA, USA) using the Single Cell 3′ v3 Library and Gel Bead Kit V3.1 (10× Genomics, #1000121, Pleasanton, CA, USA) and Chromium Single Cell G Chip Kit (10× Genomics, #1000120, Pleasanton, CA, USA) to generate single-cell gel bead-in-emulsions (GEMs) following the manufacturer’s protocol. Briefly, single cells were suspended in PBS containing 0.04% BSA. Approximately 75,981 cells were loaded per channel, with an estimated recovery of about 69,102 cells. Captured cells were lysed, and the released RNA was barcoded via reverse transcription in individual GEMs.

Reverse transcription was carried out using an S1000™ Touch Thermal Cycler (Bio-Rad, Hercules, CA, USA) under the following conditions: 53 °C for 45 min, 85 °C for 5 min, and hold at 4 °C. The synthesized cDNA was subsequently amplified, and its quality was assessed using an Agilent 4200 system (performed by CapitalBio Technology, Beijing, China).

### 2.4. Single-Cell RNA-Seq Library Preparation

Single-cell RNA-seq libraries were constructed using the Single Cell 3′ v3 Library and Gel Bead Kit V3.1 (10× Genomics, #1000121, Pleasanton, CA, USA) according to the manufacturer’s instructions. The libraries were sequenced on an Illumina NovaSeq 6000 instrument (Illumina, San Diego, CA, USA) with a PE150 strategy, achieving a minimum sequencing depth of 65,962 reads per cell (performed by CapitalBio Technology, Beijing, China).

### 2.5. Raw Data Processing, Quality Control, and Clustering

Raw sequencing data were processed using the Cell Ranger pipeline (v6.0.1) with alignment to the GRCh38 reference transcriptome. The resulting feature-barcode matrix was imported into Seurat (v4.0.0) for all downstream analyses. A standardized single-cell analysis workflow was applied as follows. Quality Control and Doublet Removal: Cells with fewer than 200 genes, a mitochondrial gene ratio >25%, or identified as potential doublets by DoubletFinder (v2.0.3; expected doublet rate = 7.5%) were filtered out. Normalization, Integration, and Clustering: Gene expression matrices were normalized and variance-stabilized using the SCTransform method. To enable a joint analysis, data from the FCD and control samples were integrated using Harmony to remove potential inter-individual batch effects. Dimensionality reduction was performed using principal component analysis (PCA). The first 30 principal components were selected for downstream analysis based on an elbow plot of standard deviations. Graph-based clustering was performed using a resolution parameter of 0.6, and results were visualized using UMAP and t-SNE. Cell Type Annotation: Clusters were annotated into major brain cell types based on the expression of canonical marker genes (e.g., AQP4 for astrocytes, C1QA for microglia, PLP1 for oligodendrocytes).

### 2.6. Differential Gene Expression Analysis and Functional Enrichment Analysis

Differential gene expression analysis across distinct cell populations was performed using the bimodal likelihood ratio test in the Seurat R package (version 4.0.0). Genes upregulated in more than 10% of cells within specific clusters were identified based on the following criteria: adjusted *p* ≤ 0.01 and absolute log2 fold change (|log2(FC)|) ≥ 0.25. To interpret the functional annotations and pathway associations of differentially expressed genes (DEGs), the following enrichment analyses were conducted:

Gene Ontology (GO) enrichment analysis: Performed using the GO database (http://geneontology.org) (accessed on 12 July 2025) to annotate DEGs with biological process, cellular component, and molecular function terms. Kyoto Encyclopedia of Genes and Genomes (KEGG) pathway analysis: Conducted using the KEGG database (https://www.genome.jp/kegg) (accessed on 12 July 2025) to identify significantly enriched signaling pathways among DEGs. DEGs meeting the significance threshold (*p* < 0.01) were ranked by |log2(FC)| in descending order for functional and pathway annotation. The complete statistical results of the functional enrichment analyses (KEGG and GO), including *p*-values, adjusted *p*-values, and gene ratios, are provided in Supplementary Tables S4–S6.

### 2.7. Statistical Analysis

Normally distributed variables are presented as the mean ± standard deviation (SD) and were compared using the unpaired student’s *t*-test. A *p*-value < 0.05 was considered statistically significant. GraphPad Prism 10 (San Diego, CA, USA) was used for generating figures and performing statistical analyses.

## 3. Results

### 3.1. Baseline Characteristics of Patients

Cortical tissue samples were obtained during neurosurgery from two patients: one 22-year-old male with drug-resistant focal cortical dysplasia (FCD) type II and one 31-year-old control male with a ventricular occupying lesion and no history of epilepsy or antiepileptic drug use (see Section 2.1 for full clinical details). The study was approved by the institutional ethics committee, and written informed consent was obtained from both participants.

### 3.2. Single-Cell Transcriptomic Profiling and Quality Control

We performed droplet-based single-cell RNA sequencing (scRNA-seq) on the collected tissues. After rigorous quality control and filtering, we retained a high-quality transcriptomic dataset of 15,942 single cells (7762 from the FCD sample and 8180 from the control) for subsequent analysis. The sequencing data exhibited high quality, with a Q30 base rate above 91.6% and over 95.6% of reads uniquely mapping to the GRCh38 reference genome, confirming the reliability of our dataset (Table 2).

### 3.3. Cell Type Identification and Heterogeneity

**Figure 1:**
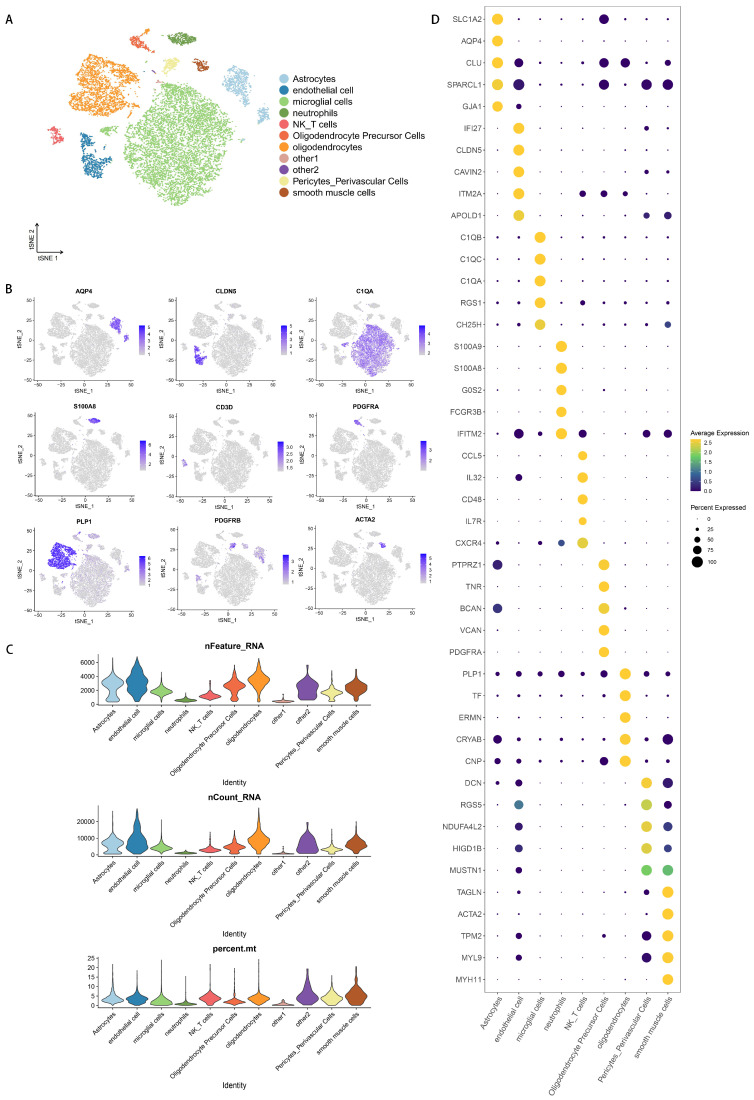
Cell clustering and marker gene identification. (**A**) t-SNE clustering of snRNA-seq data. Cell types were annotated to the expression of known marker genes and exhibited by t-SNE visualization with the same cells merged. (**B**) The expression of classic marker genes displayed by a tSNE plot. Each dot represents a cell. The darker the dot, the higher the marker gene expression in a specific cell. (**C**) Distribution of genes and UMIs per cell across all identified clusters, demonstrating the sequencing depth and data quality. (**D**) A bubble chart displaying the top five genes in each cluster.

Unsupervised clustering of the integrated scRNA-seq data, derived from whole cell suspensions of fresh tissue, revealed 11 distinct non-neuronal cell populations (Figure 1A). These clusters were annotated based on the expression of well-established canonical marker genes: AQP4 (astrocytes), CLDN5 (endothelial cells), C1QA (microglia), S100A8 (neutrophils), CD3D (NK/T cells), PDGFRA (oligodendrocyte precursor cells, OPCs), PLP1 (oligodendrocytes), PDGFRB (pericytes), and ACTA2 (smooth muscle cells) (Figure 1B). The transcriptomic diversity and sequencing depth across all clusters were uniform, as visualized by the distribution of genes and UMIs per cell (Figure 1C). The top five marker genes for each cluster are displayed in a bubble plot (Figure 1D).

### 3.4. Dramatic Reconstitution of the Glial and Vascular Ecosystem in FCD

**Figure 2:**
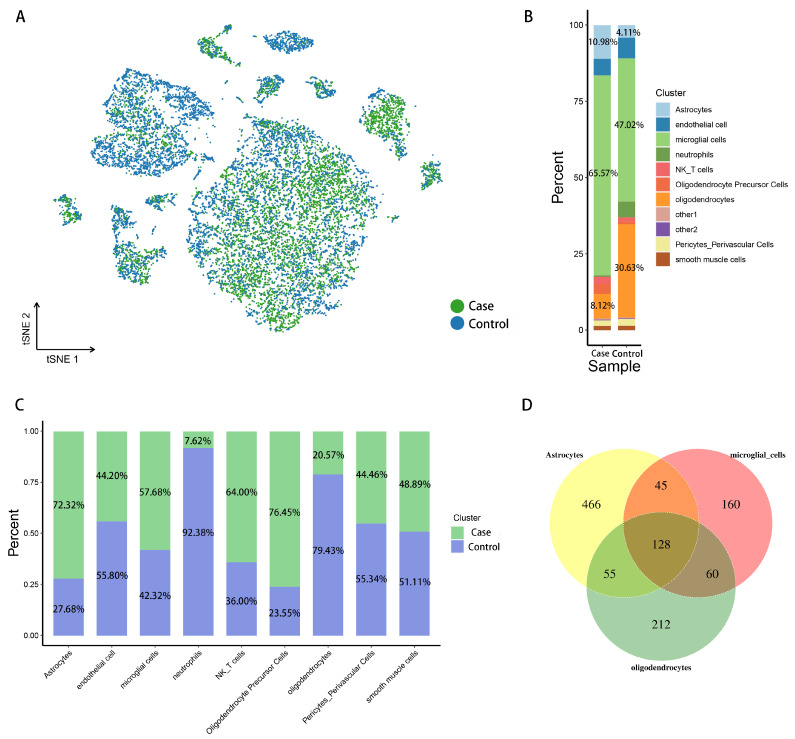
Cell clusters were differentially distributed in two groups. (**A**,**B**) show the number of cells by tSNE and a bar chart. (**C**) Bar chart showing the detailed ratio of cells in samples. (**D**) A Venn chart of the DEGs in three clusters. A total of 128 genes were co-expressed in three clusters.

A comparative analysis revealed significant alterations in both the cellular composition and intrinsic transcriptional states within the FCD cortex. We observed a notable expansion of microglia (65.57% vs. 47.02% of all cells; approximately a 39% relative increase) and astrocytes (10.98% vs. 4.11%; approximately a 167% relative increase), alongside a substantial loss of oligodendrocytes (8.12% vs. 30.63%; approximately a 73% relative decrease) (Figure 2A–C). As expected from our whole-cell sequencing approach, neuronal populations were not captured; thus, our analysis concentrated on the reconstitution of the glial and vascular compartments. The proportions of other captured cell types, including endothelial cells, T cells, fibroblasts, monocytes, and OPCs, remained relatively stable between the two groups.

Importantly, in addition to these shifts in cellular abundance, we identified extensive intracellular transcriptomic reprogramming within the glial populations. This was evidenced by the substantial number of differentially expressed genes (DEGs) identified within each of the altered glial types: 694 DEGs in astrocytes, 393 in microglia, and 455 in oligodendrocytes (Supplementary Table S1). This dual phenomenon of population-level expansion or loss, coupled with cell-state-level molecular alterations, highlights a profound glial-centric reorganization of the FCD microenvironment.

### 3.5. Identification of a Core Set of Differentially Expressed Genes Across Glial Cells

To investigate the molecular underpinnings of these changes, we identified differentially expressed genes (DEGs) within the three major glial populations. We found 694 DEGs in astrocytes (355 up, 339 down), 393 DEGs in microglia (72 up, 321 down), and 455 DEGs in oligodendrocytes (23 up, 432 down) (Supplementary Table S1). Notably, a core set of 128 DEGs was shared across all three glial cell types (Figure 2D), indicating convergent pathological mechanisms despite their divergent cellular fates. Among these, RAC1 was consistently upregulated, while ATP5F1D was consistently downregulated, pointing to potential roles in pro-inflammatory activation and mitochondrial dysfunction, respectively (Supplementary Tables S1–S3).

### 3.6. Functional Enrichment Analysis Reveals Convergent Neuroinflammatory Pathways

**Figure 3:**
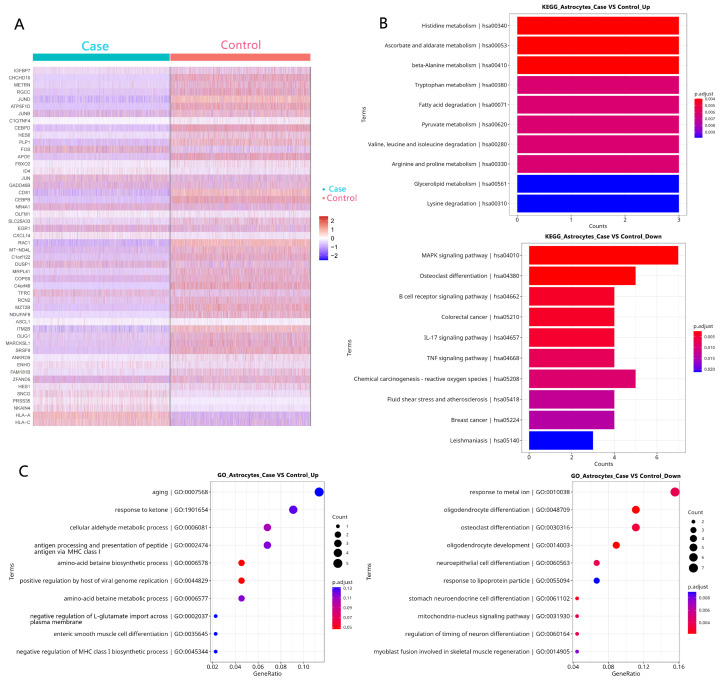
DEG identification and function analysis in astrocytes. (**A**) A heatmap of the top 50 DEGs in astrocytes. (**B**) The KEGG enrichment of DEGs in astrocytes. (**C**) A GO analysis of DEGs in astrocytes.

**Figure 4:**
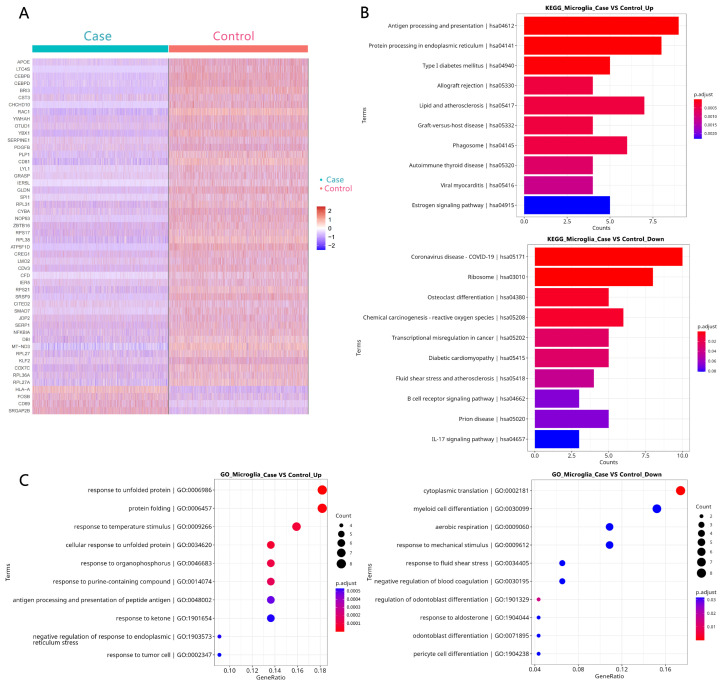
DEG identification and function analysis in microglia. (**A**) A heatmap of the top 50 DEGs in microglia. (**B**) The KEGG enrichment of DEGs in microglia. (**C**) A GO analysis of DEGs in microglia.

**Figure 5:**
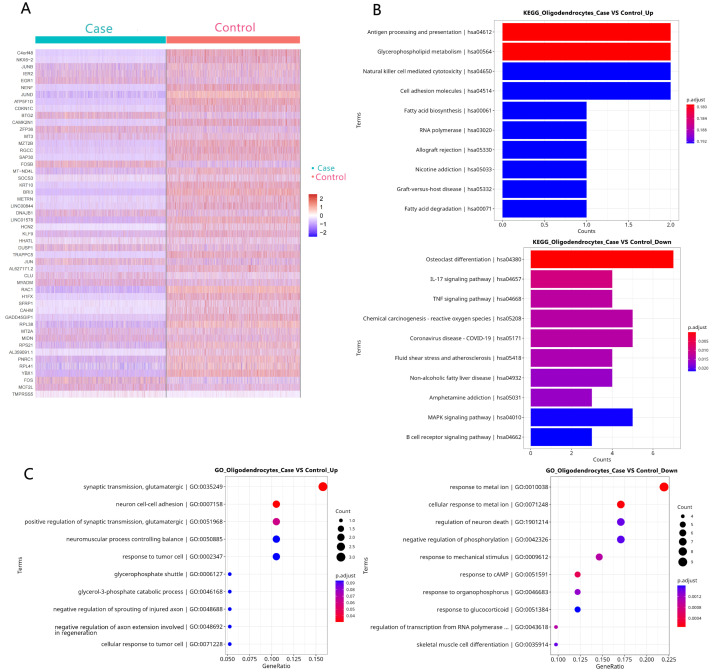
DEG identification and function analysis in oligodendrocytes. (**A**) A heatmap of the top 50 DEGs in oligodendrocytes. (**B**) The KEGG enrichment of DEGs in oligodendrocytes. (**C**) A GO analysis of DEGs in oligodendrocytes.

GO and KEGG pathway analyses were performed on the DEGs from each glial population (Figure 3, Figure 4 and Figure 5). Strikingly, the shared pathogenic signature was reflected in the enrichment of common signaling pathways. Five KEGG pathways were significantly enriched across astrocytes, microglia, and oligodendrocytes, most notably the IL-17 signaling pathway and osteoclast differentiation (Figure 3B, Figure 4B and Figure 5B). This highlights a coordinated, cross-glial engagement of potent neuroinflammatory networks in the FCD epileptic focus.

### 3.7. Attenuated Cell–Cell Communication in the FCD Microenvironment

**Figure 6:**
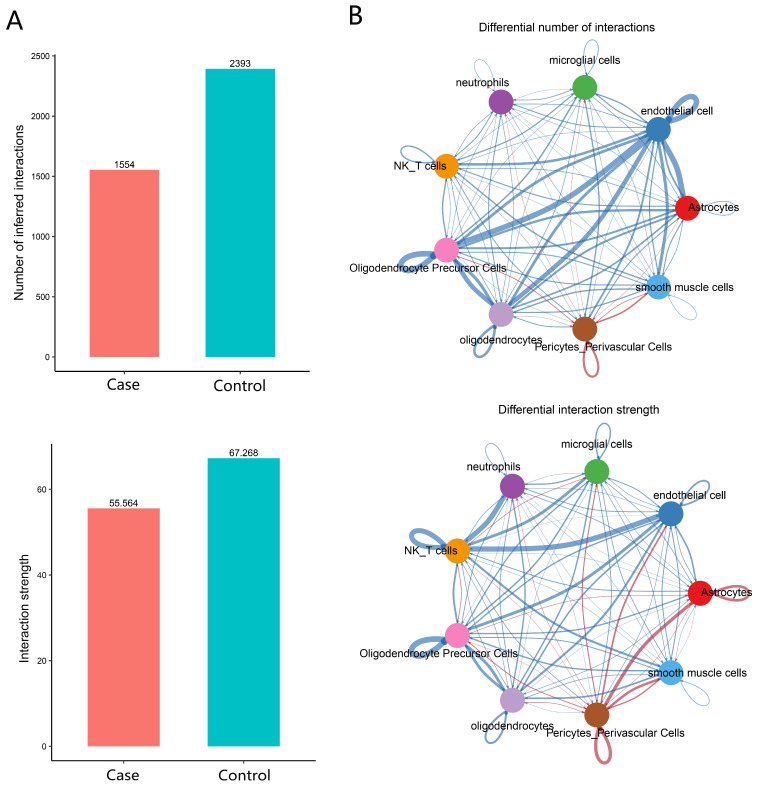
Comparative analysis of intercellular communication networks. (**A**) Bar plot showing the total number and strength of interactions in FCD and control groups. (**B**) Differential circle plot depicting altered ligand–receptor interactions. Edge width represents the number of interactions; red and blue colors indicate signaling increased and decreased in FCD compared to the control, respectively.

To assess the functional consequences of the cellular and transcriptional alterations, we inferred intercellular communication networks among the captured glial and vascular populations using CellChat. The overall signaling activity was significantly weakened in FCD tissue. The total number of inferred interactions decreased by 35% (FCD: 1554 vs. Control: 2393), and the overall interaction strength was lower (FCD: 55.56 vs. Control: 67.27) (Figure 6A). Analysis of signaling pathways revealed a broad attenuation in FCD. The COMPLEMENT and MHC-I pathways exhibited the most pronounced reductions in information flow, alongside other significantly downregulated pathways including GALECTIN, SEMA4, and ANNEXIN (Supplementary Figure S1). At the ligand–receptor level, differential analysis identified a predominant weakening of interactions originating from microglia. Specifically, the complement interaction C3–C3AR1 from microglia to astrocytes and oligodendrocytes was markedly reduced. Additionally, CX3CL1–CX3CR1 signaling between microglia and other glial cells was significantly attenuated. Among the most affected astrocytic interactions was the reduction in NRXN1–NLGN1 signaling (Supplementary Figure S2). Overall, microglia demonstrated the most substantial decrease in outgoing communication strength, while astrocytes exhibited a more mixed pattern characterized by a general reduction in signaling but relative preservation of some specific interactions (Supplementary Figures S1 and S2). This global attenuation indicates a widespread disruption of coordinated cellular crosstalk within the FCD microenvironment.

## 4. Discussion

### 4.1. A Reconstituted Glial Ecosystem in FCD Characterized by Microgliosis, Astrogliosis, and Oligodendrocyte Loss

In this study, we constructed the first single-cell transcriptomic atlas of human cortical tissue from a surgical patient with FCD type II drug-resistant epilepsy and a matched control. Our analysis revealed a profoundly reconstituted cellular ecosystem characterized by glial-specific molecular alterations that converge on pro-inflammatory and bioenergetic pathways, providing novel insights into the mechanisms of epileptogenesis in FCD.

The most striking finding is the dramatic reconstitution of the glial landscape. We observed a significant expansion of microglia (65.57% vs. 47.02%) and astrocytes (10.98% vs. 4.11%) alongside a marked loss of oligodendrocytes (8.12% vs. 30.63%) in the FCD lesion (Figure 2C). This triad illustrates a hyper-inflammatory and dysmature tissue environment, consistent with previous histopathological reports of gliosis and myelination defects in FCD [20]. However, unlike traditional bulk RNA sequencing, our study delineates the specific alterations within individual glial cell types at single-cell resolution, thereby providing higher-resolution evidence for the cellular pathology of FCD. Microglial expansion is a well-documented feature of epileptic foci, driving neuroinflammation through the release of cytokines and phagocytic activity [14,21]. Our findings strongly support and extend this notion: in FCD, the DEGs in microglia were significantly enriched in inflammatory pathways (Figure 4B), suggesting that their activation may directly or indirectly provoke neuronal hyperexcitability via the release of pro-inflammatory factors such as IL-1β and TNF-α. This aligns with the view proposed by Vezzani et al. that “microglia-driven inflammation is central to the maintenance of the epileptic focus” [22]. Similarly, astrogliosis can lead to the loss of homeostatic functions and a gain of pro-inflammatory properties, further exacerbating neuronal hyperexcitability [23]. The observed expansion of astrocytes in our FCD sample holds significant pathological implications. Normally, astrocytes play a crucial role in regulating the neural microenvironment by uptaking synaptic glutamate and maintaining the integrity of the blood–brain barrier. Their aberrant proliferation in FCD likely disrupts ionic homeostasis and neurotransmitter clearance, thereby facilitating the formation of an epileptic focus [3,4]. Furthermore, the upregulation of antigen-presentation-related genes (e.g., HLA-A, HLA-C) in astrocytic DEGs (Table 3) suggests a potential role in amplifying the neuroinflammatory response through enhanced immune activation, a finding corroborated by our GO enrichment analysis.

The significant loss of oligodendrocytes, a key finding of our study, indicates not only a disruption in axonal integrity but also a potential failure in OPC differentiation, a process that is strongly inhibited by inflammatory cytokines [3]. Our single-cell data further elucidate this understanding by revealing that oligodendrocytic DEGs are significantly enriched in myelination-related pathways as well as in processes critical to inflammation and energy metabolism. The upregulation of pathways such as Natural Killer cell-mediated cytotoxicity and antigen processing and presentation (Figure 5B) highlights a robust adaptive immune response, which is a well-established hallmark of FCD tissue [24]. Conversely, the downregulation of key metabolic processes indicates a broad suppression of cellular homeostasis. Importantly, this observed bioenergetic deficit supports the notion that oligodendrocytes are essential metabolic supporters of axons, and their impairment can lead to neuronal network instability [25]. The interplay between inflammatory signaling and metabolic failure may exacerbate FCD pathology through a multifaceted mechanism, strongly supported by clinical evidence linking mTOR-driven defects in oligodendroglial turnover and maturation to myelin deficiency in FCD and TSC [26]. Thus, our findings provide a novel molecular basis for the pharmacoresistance observed in FCD patients, positioning glial dysfunction, which encompasses dysregulated inflammation, impaired differentiation, and bioenergetic failure, as a potential central contributor to epileptogenesis in developmental disorders.

### 4.2. Convergent Molecular Pathways Across Glial Cells Highlight Shared Mechanisms of Inflammation and Energetic Deficit

Beyond cellular reconstitution, we unveiled a core set of 128 DEGs shared among microglia, astrocytes, and oligodendrocytes. This finding indicates that despite their distinct cellular fates, these glial populations are co-opted into a common pathogenic program in FCD. Notably, the consistent upregulation of RAC1 and downregulation of ATP5F1D are particularly significant. RAC1 is a Rho GTPase that acts as a central regulator of pro-inflammatory signaling, cytoskeletal reorganization, and phagocytosis in glial cells [27,28]. Its coordinated upregulation across all three glial types underscores a unified state of immune activation. Conversely, the downregulation of ATP5F1D, a key subunit of mitochondrial ATP synthase, indicates a pervasive disruption in oxidative phosphorylation and bioenergetic capacity [29,30]. We hypothesize that RAC1-mediated inflammation and ATP5F1D-linked mitochondrial dysfunction create a vicious cycle: inflammation depletes energy and damages mitochondria, while energy failure exacerbates inflammatory signaling and hinders cellular functions essential for tissue repair and homeostasis.

### 4.3. Functional Enrichment Implicates the IL-17 Signaling Pathway as a Central Hub in Glial Dysregulation

This convergent molecular pathology is further reflected in the functional enrichment analysis. The shared enrichment of the IL-17 signaling pathway across all three glial types represents a pivotal discovery. The IL-17 pathway is a potent driver of inflammatory responses in neurological diseases [20]. Its involvement in FCD suggests a previously underappreciated role for this specific inflammatory axis in epileptogenesis. Additionally, other shared pathways, such as osteoclast differentiation, may metaphorically reflect enhanced phagocytic and tissue-destructive capabilities within the brain lesion.

### 4.4. Attenuated Intercellular Communication and Its Etiological Implications in FCD

Our CellChat analysis revealed a significant attenuation of intercellular communication within the FCD epileptic microenvironment, characterized by a 35% reduction in the number of inferred interactions and a decrease in overall interaction strength (Figure 6A). This attenuation predominantly originated from key immune cells such as microglia, neutrophils, and NK/T cells, while astrocytes exhibited a heterogeneous pattern of reprogramming (Figure 6B), indicating a systematic breakdown of coordinated cellular crosstalk.

The observed attenuation of cell–cell communication in FCD contrasts sharply with the enhanced inflammatory signaling typical of temporal lobe epilepsy with hippocampal sclerosis (TLE-HS). In TLE-HS, an acquired condition, chronic neuroinflammation promotes robust pro-inflammatory communication, evidenced by the upregulation of mediators such as Cox2 and Cxcl10 and the activation of TNF and IL-17 pathways [31]. In contrast, FCD, which is a developmental disorder, primarily stems from PI3K-mTOR mutations in neuroectodermal progenitors. This leads to intrinsic network construction deficits without significant inflammation [19]. The principal reason for this discrepancy lies in the etiological distinction, where one condition arises from a developmental defect and the other from an acquired inflammatory adaptation.

Methodological differences also contribute to this contrast. Our use of CellChat emphasizes strong, biologically relevant interactions, highlighting the global attenuation in FCD. Some TLE-HS studies utilizing CellPhoneDB may capture a broader range of potential inflammatory pairs through permutation tests [19]. Rigorous quality control in both our study and key TLE-HS research [32] ensures that these differences reflect true biology rather than technical artifacts.

Our conclusion regarding attenuated communication is strongly supported by recent single-cell studies on FCD. Galvão et al. [18] demonstrated that impaired microglial-neuronal crosstalk, evidenced by the downregulation of communication-related genes such as CD47 and CX3CR1, contributes to epileptogenesis in FCD II. This finding aligns perfectly with our observed reduction in microglial signaling output. Furthermore, Galvão et al. [33] reported astrocytic reprogramming in FCD, where some subpopulations acquired pro-inflammatory features while others lost neuroprotective functions. This explains the heterogeneous pattern of both the increased and decreased communication interactions that we observed for astrocytes. Most directly, Bizzotto et al. [19], also utilizing CellChat, confirmed the widespread attenuation of intercellular signaling networks in FCD, including reduced activity in specific epilepsy-related ligand–receptor pairs such as TNF-TNFR1 and IL-1β-IL-1R1.

### 4.5. Positioning Within the Single-Cell Landscape of Epileptic Cortex

Recent single-cell and single-nucleus transcriptomic studies have significantly advanced our understanding of the cellular underpinnings of the epileptic cortex. For instance, a multimodal study on FCD type IIa highlighted the vulnerability of excitatory neurons and the activated ion of oligodendrocyte precursor cells (OPCs), alongside aberrant neuron-OPC crosstalk [34]. While such studies have been instrumental, they primarily focus on neuronal compartments or specific glial subtypes. Our study provides a distinct and complementary vantage point by revealing a coordinated glial dysregulation intrinsic to FCD type II.

It is increasingly recognized that the normal neocortex possesses a conserved cellular architecture with precise regional specializations in glial subtypes and an excitatory-inhibitory balance, which are fundamental for local circuit function [35]. In FCD type II, we observed a profound disruption of this delicate cellular blueprint. In contrast to the neuron-OPC axis emphasized in FCD IIIa [34], we demonstrate that astrocytes, microglia, and oligodendrocytes in FCD II share a core set of 128 differentially expressed genes. This finding reveals a convergent pathogenic mechanism wherein pro-inflammatory activation (e.g., via RAC1/IL-17 signaling) is coupled with mitochondrial metabolic failure (e.g., via ATP5F1D downregulation) across this tripartite glial axis—a phenomenon not previously reported. This glia-centric perspective complements and extends prior work in FCD that described distinct microglial and immature astrocytic states [18] by uncovering the shared molecular disruption that unifies these glial populations.

Our analysis of cell–cell communication revealed a significant and widespread attenuation of signaling, particularly from microglia (e.g., COMPLEMENT and CX3CL1-CX3CR1 axes), as well as involving MHC-I pathways. This “developmental disconnection” phenotype, characterized by a breakdown in coordinated glial crosstalk, contrasts with the specific neuron-OPC communication deficits reported in FCD IIIa and highlights a distinct pathophysiological mechanism in FCD type II [34]. While Galvão et al. [18] noted upregulated MHC-II signaling in specific microglial subsets, our observation of a global attenuation in microglial signaling output and MHC-I-related communication indicates a more pervasive failure of immune coordination within the FCD II microenvironment.

By integrating inflammatory, metabolic, and communicative deficits into a unified, glia-centric framework, our study addresses a critical gap in the existing literature. We advance beyond merely characterizing isolated cell states to unveil a convergent glial pathology that offers a novel and coherent explanation for network hyperexcitability in this developmental disorder.

### 4.6. Technical Limitations and Validation Strategies

Our study presents several technical limitations that must be acknowledged when interpreting the results, alongside clear strategies for their resolution in future research. First, while the use of whole-cell RNA sequencing from fresh tissue is advantageous for transcript capture efficiency, it has resulted in the underrepresentation of vulnerable neuronal populations. This inherent technical bias restricts our findings primarily to the glial and vascular compartments. To address this limitation, future studies should employ single-nucleus RNA sequencing on frozen tissue archives to capture the full cellular diversity of the epileptic focus. Second, the exploratory nature of our study, which involves a single focal cortical dysplasia (FCD) case and control, limits both the statistical power and generalizability of our findings. Therefore, robust validation in larger, independent patient cohorts is essential, including orthogonal confirmation of key transcriptomic alterations using methods such as quantitative PCR (qPCR) and immunohistochemistry. Finally, our functional interpretations of the identified pathways remain descriptive and correlative. Thus, the proposed roles of RAC1 and IL-17 signaling in FCD pathogenesis require functional validation through targeted interventions in relevant in vitro or in vivo models. Despite these technical constraints, this exploratory profile provides a valuable resource and generates specific, testable hypotheses, establishing a rigorous foundation for future mechanistic and translational studies.

Furthermore, in the interest of scientific rigor, we attempted to validate key differentially expressed genes (RAC1, IL-17, ATP5F1D) using qPCR on the extremely limited remaining tissue samples. The results were mixed: ATP5F1D exhibited significant downregulation, providing supportive evidence for our scRNA-seq findings. RAC1 demonstrated a consistent upregulatory trend but did not achieve statistical significance. In contrast, the mRNA level of IL-17 displayed a discordant trend (see Supplementary Figure S3). These divergent outcomes likely arise from several factors, including inherent methodological differences between single-cell and bulk transcriptomic assays, the fact that IL-17 signaling is primarily mediated at the protein level, and most critically, the substantial inter-individual variability and lack of statistical power due to our minimal sample size (*n* = 1). We provide these validation data in the Supplementary Materials to ensure full transparency and emphasize that while they offer partial support for certain pathways, they collectively highlight the necessity for future validation in expanded cohorts.

### 4.7. Strategic Outlook: A SWOT Analysis of Glial Dysregulation in FCD

A SWOT (Strengths, Weaknesses, Opportunities, Threats) analysis provided a structured framework for critically evaluating our study and delineating its strategic implications for future research.

Strengths: This study presents the first unbiased, high-resolution transcriptomic atlas specifically focused on the glial and vascular compartments in FCD type II, utilizing whole-cell single-cell RNA sequencing (scRNA-seq) from fresh surgical tissue. We advance beyond merely characterizing isolated cell states to uncover a convergent glial pathology, revealing a core set of shared inflammatory and metabolic disruptions across astrocytes, microglia, and oligodendrocytes. The integrated analysis, which combines differential expression, pathway enrichment, and cell–cell communication inference, offers a comprehensive systems-level view of the FCD microenvironment.

Weaknesses: As detailed in Section 4.6, the primary limitations include the exploratory nature of the study design with a limited cohort, the technical bias of whole-cell sequencing against neuronal populations, and the descriptive nature of the transcriptomic findings.

Opportunities: The identified convergent pathways, such as RAC1 and IL-17 signaling, represent compelling, hypothesis-driven targets for future mechanistic studies in experimental models. Our dataset serves as a foundational resource for validating key findings in larger cohorts, particularly through the application of single-nucleus RNA sequencing on frozen tissue archives to include neurons. This approach opens avenues for multi-omics integration, such as with proteomics or spatial transcriptomics, to further elucidate the molecular architecture of FCD lesions.

Threats to Validity: There are potential, albeit minimal, confounding effects arising from the underlying ventricular pathology of control patients. Furthermore, the intrinsic technical and biological variability associated with single-cell genomics may influence the reproducibility of specific low-abundance transcripts. The translational pathway from identifying actionable pathways to developing effective therapies for developmental disorders is both lengthy and complex.

This SWOT analysis underscores that, despite inherent limitations, the strengths of our study in generating novel, high-quality hypotheses provide significant opportunities to deepen our understanding of FCD pathophysiology and establish a foundation for future translational research.

## 5. Conclusions

This exploratory study presents a comprehensive single-cell transcriptomic profiling of human cortical tissue from a patient with FCD type II epilepsy. We identified a reconstituted glial ecosystem characterized by the expansion of microglia and astrocytes, alongside a significant loss of oligodendrocytes. A core set of 128 differentially expressed genes was identified across glial cell types, including upregulated RAC1 and downregulated ATP5F1D, indicating convergent mechanisms of neuroinflammation and mitochondrial dysfunction.

Functional enrichment analyses further underscored the IL-17 signaling pathway as a central hub in glial dysregulation. Moreover, cell–cell communication was significantly attenuated in the FCD microenvironment, suggesting a breakdown in coordinated intercellular crosstalk. These findings provide a foundational resource for understanding the pathophysiology of FCD at a cellular resolution and highlight RAC1 and IL-17 signaling as key candidates for future therapeutic exploration. Future research should focus on validating these mechanisms in larger cohorts and functional models, integrating multi-omics approaches, and correlating molecular profiles with clinical outcomes to pave the way for targeted therapies in drug-resistant FCD epilepsy.

## Figures and Tables

**Table 1:** Methodological landscape of single-cell studies in epilepsy and focal cortical dysplasia.

Study (Year)	Disease Focus	Methodological Approach	Key Findings	Novelty & Contribution	Context for This Study
Kumar et al., 2022 [14]	Various Focal Epilepsies	CITE-seq on human surgical epileptic lesions.	Identified pro-inflammatory T cell–microglia immune complexes.	First demonstration of direct, pro-inflammatory immune cell interactions in human epilepsy	Highlights neuroinflammation, but not FCD-specific glial ecology
Wen et al., 2024 [17]	Post-traumatic vs. Hereditary Epilepsy	Comparative scRNA-seq of cellular heterogeneity across epilepsy etiologies.	Distinct cellular compositions and inflammatory pathways between epilepsy types.	Systematic cross-etiology comparison of cell-type-specific signatures	Provides a framework for subtype investigation, but not specific to MCDs like FCD
Galvão et al., 2024 [18]	FCD	Multimodal single-nucleus sequencing (snRNA-seq + snATAC-seq) of human FCD tissue	Revealed neuronal vulnerability, dysmorphic neurons, and activated microglia/astrocytes	Uncovered coordinated transcriptomic and epigenomic alterations in FCD	Directly characterizes FCD pathology, but focuses on neuronal compartments
Bizzotto et al., 2025 [19]	Mosaic Focal Epilepsies	Single-cell genotyping-transcriptome co-profiling of human tissue with somatic mutations	Dissected cell-autonomous (e.g., mutant neurons) vs. non-cell-autonomous (e.g., microglia activation) effects	Pioneered methods to disentangle cell-autonomous vs. non-cell-autonomous disease mechanisms	Reveals non-cell-autonomous glial responses, underscoring the need for systematic glial studies

Abbreviations: FCD, Focal Cortical Dysplasia; MCDs, Malformations of Cortical Development; scRNA-seq, Single-Cell RNA Sequencing; snRNA-seq, Single-Nucleus RNA Sequencing; snATAC-seq, Single-Nucleus Assay for Transposase-Accessible Chromatin with high-throughput sequencing; CITE-seq, Cellular Indexing of Transcriptomes and Epitopes by Sequencing.

**Table 2:** Basic sequencing information.

Sample	FCD	Control
Summary of raw data volume		
Raw data (Gb)	201.52	161.87
Raw Reads	671,721,934	539,567,458
Fraction of Reads Kept	83.7%	100.0%
Data quality control statistics		
Valid Barcodes	97.8%	97.8%
Q30 Bases in Barcode	96.7%	96.3%
Q30 Bases in RNA Read	91.6%	92.9%
Q30 Bases in UMI	96.2%	96.0%
Comparison Statistics		
Reads Mapped to Genome	96.6%	95.6%
Reads Mapped Confidently to Genome	94.0%	90.2%
Reads Mapped Confidently to Intergenic Regions	5.7%	5.7%
Reads Mapped Confidently to Intronic Regions	44.5%	35.1%
Reads Mapped Confidently to Exonic Regions	43.8%	49.4%
Reads Mapped Confidently to Transcriptome	40.3%	45.6%
Reads Mapped Antisense to Gene	1.8%	1.9%
Cell number correlation statistics		
Barcode Count in Whitelist	1,819,154	1,892,085
Detected Barcode Count	1,120,822	1,233,143
Detected Barcode UMI Count	39,508,383	68,413,897
Estimated Barcode Count	7762	8180
Estimated Barcode UMI Count	34,707,780	58,564,816
Summary of expression statistics		
Mean Reads per Cell	86,539	65,961
Median UMI Counts per Cell	4011.5	5919
Sequencing Saturation	85.2%	71.7%
Total Genes Detected	26,124	26,525
Median Genes per Cell	1817.5	2297.5

Abbreviations: FCD, Focal cortical dysplasia epilepsy sample; Con, Contral Sample.

**Table 3:** List of the top five upregulated and downregulated genes in three clusters.

Astrocytes		Microglia		Oligodendrocyte	
Gene Name	Log FC	Gene Name	Log FC	Gene Name	Log FC
SNCG	1.086259848	HLA-A	0.561057014	MCF2L	0.53631139
PRSS35	0.879550974	FOSB	0.555225766	TMPRSS5	0.526303683
NKAIN4	0.626660741	CD69	0.548330447	AL033523.1	0.466653227
HLA-A	0.604728496	SRGAP2B	0.533151767	KIAA0930	0.44636579
HLA-C	0.562904225	ARHGAP24	0.514877263	STMN4	0.398002199
IGFBP7	−1.14329361	APOE	−1.282753784	C4orf48	−1.270132423
CHCHD10	−1.107841152	LTC4S	−1.227630496	NKX6−2	−1.043764641
METRN	−1.080788609	CEBPB	−1.202798566	JUNB	−0.940319648
RGCC	−1.078778253	CEBPD	−1.034718416	IER2	−0.855560116
JUND	−1.054184776	BRI3	−0.906932045	EGR1	−0.847209539

## Data Availability

The data presented in this study are available on request from the corresponding author. The data are not publicly available due to privacy/ethical restrictions.

## Supplementary Materials

The following supporting information can be downloaded at: https://www.mdpi.com/article/10.3390/biology14121690/s1, Supplementary Table S1: DEGs in Astrocytes; Supplementary Table S2: DEGs in Microglia; Supplementary Table S3: DEGs in Oligodendrocytes; Supplementary Table S4: KEGG and GO enrichment_Top 10 up and downregulated pathways in Astrocytes; Supplementary Table S5: KEGG and GO enrichment_Top 10 up and downregulated pathways in Mgliaicro; Supplementary Table S6: KEGG and GO enrichment_Top 10 up and downregulated pathways in Oligodendrocytes; Supplementary Figure S1: Comparison_Interaction_Relative_information_flow; Supplementary Figure S2: Comparison_Interaction_Bubble; Supplementary Figure S3: Relative mRNA level of RAC1, IL17, ATP5F1D.

## Abbreviations

The following abbreviations are used in this manuscript:

Abbreviation	Full Name
FCD	Focal Cortical Dysplasia
scRNA-seq	Single-Cell RNA Sequencing
DEGs	Differentially Expressed Genes
GO	Gene Ontology
KEGG	Kyoto Encyclopedia of Genes and Genomes
MCDs	Malformations of Cortical Development
TSC	Tuberous Sclerosis Complex
SUDEP	Sudden Unexpected Death in Epilepsy
TWAS	Transcriptome-Wide Association Study
PWAS	Proteome-Wide Association Study
ceRNA	Competing Endogenous RNA
RNA-Seq	RNA Sequencing
PBS	Phosphate-Buffered Saline
GEMs	Gel Bead-in-Emulsions
BSA	Bovine Serum Albumin
cDNA	Complementary DNA
UMI	Unique Molecular Identifier
PCA	Principal Component Analysis
t-SNE	t-Distributed Stochastic Neighbor Embedding
UMAP	Uniform Manifold Approximation and Projection
SD	Standard Deviation
OPCs	Oligodendrocyte Precursor Cells
mTOR	Mechanistic Target of Rapamycin
PI3K	Phosphoinositide 3-Kinase
TLE-HS	Temporal Lobe Epilepsy with Hippocampal Sclerosis
IL-17	Interleukin-17
RAC1	Ras-related C3 botulinum toxin substrate 1
ATP5F1D	ATP Synthase F1 Subunit Delta
IL-1β	Interleukin-1 Beta
TNF-α	Tumor Necrosis Factor Alpha
NRXN1	Neurexin 1
NLGN1	Neuroligin 1
CX3CL1	Chemokine (C-X3-C motif) ligand 1
CX3CR1	Chemokine (C-X3-C motif) receptor 1
C3	Complement C3
C3AR1	Complement C3a receptor 1
