# Supplementary figures and images for: Exploratory Single-Cell Transcriptomic Profiling Reveals Dysregulated Glial Populations and Pathways in Focal Cortical Dysplasia Epilepsy
Source: Biology (Basel). 2025 Nov 27;14(12):1690. doi: 10.3390/biology14121690 (PMC12730583; doi:10.3390/biology14121690)

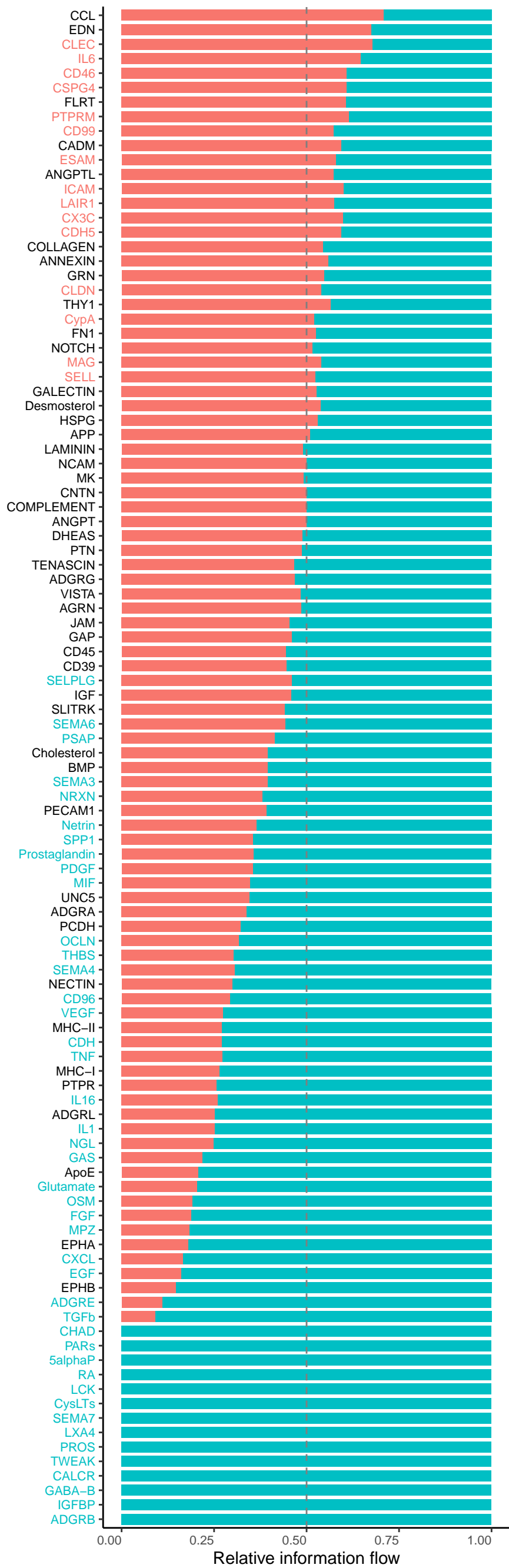

brain1102  
brain0926

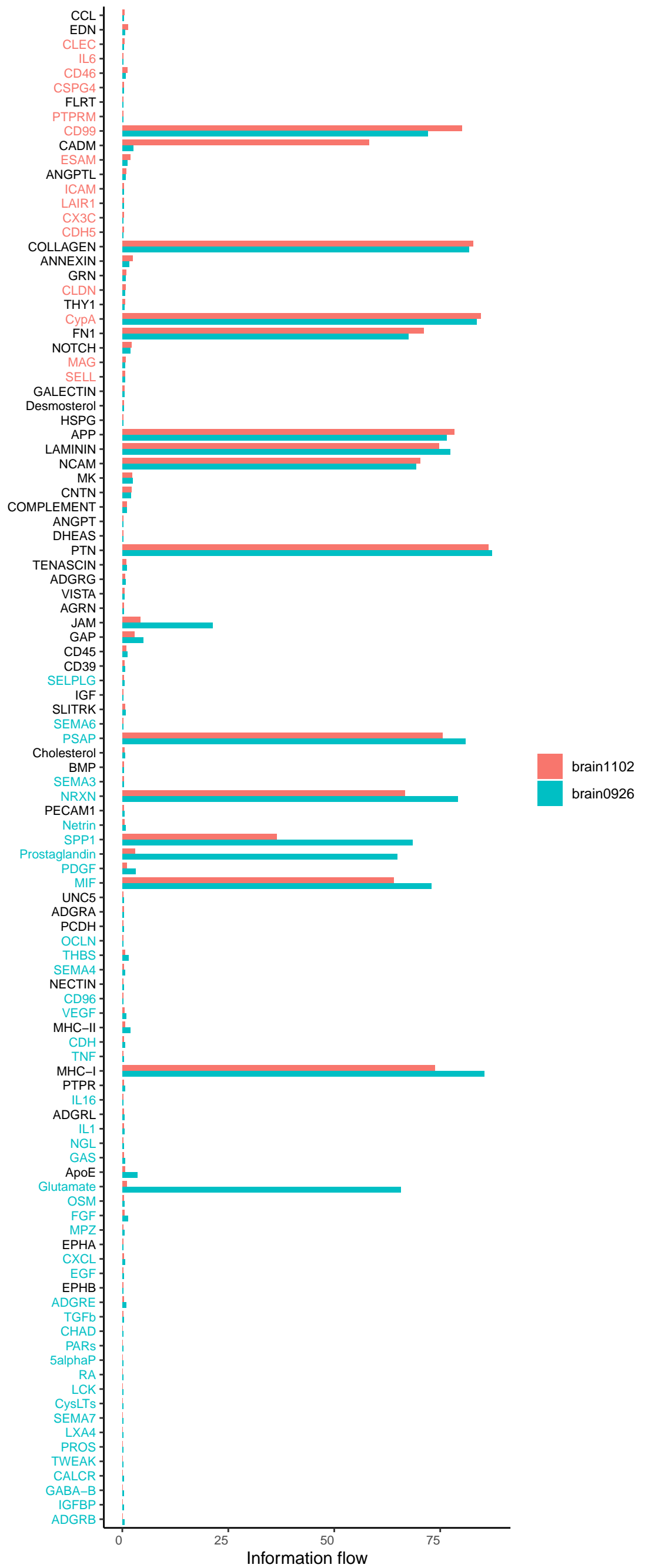

brain1102  
brain0926

Supplement: Supplementary file 1 [file biology-14-01690-s001.zip › Supplementary Figure S1.Comparison_Interaction_Relative_information_flow.pdf]

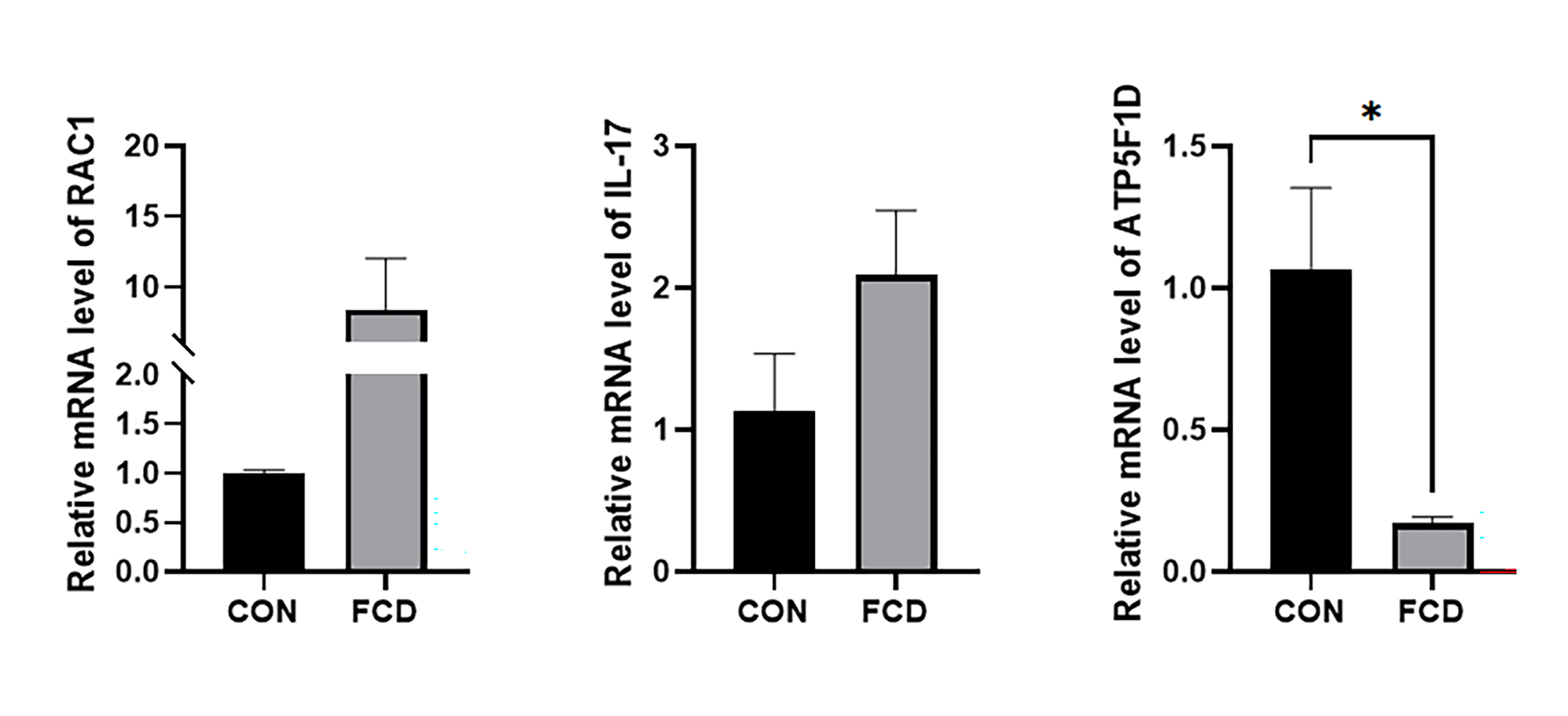

Supplement: Supplementary file 1 [file biology-14-01690-s001.zip › Supplementary Figure S3 - Relative mRNA level of RAC1,IL17,ATP5F1D.png]
