# Supplementary material for: Exploratory Single-Cell Transcriptomic Profiling Reveals Dysregulated Glial Populations and Pathways in Focal Cortical Dysplasia Epilepsy
Source: Biology (Basel). 2025 Nov 27;14(12):1690. doi: 10.3390/biology14121690 (PMC12730583; doi:10.3390/biology14121690)

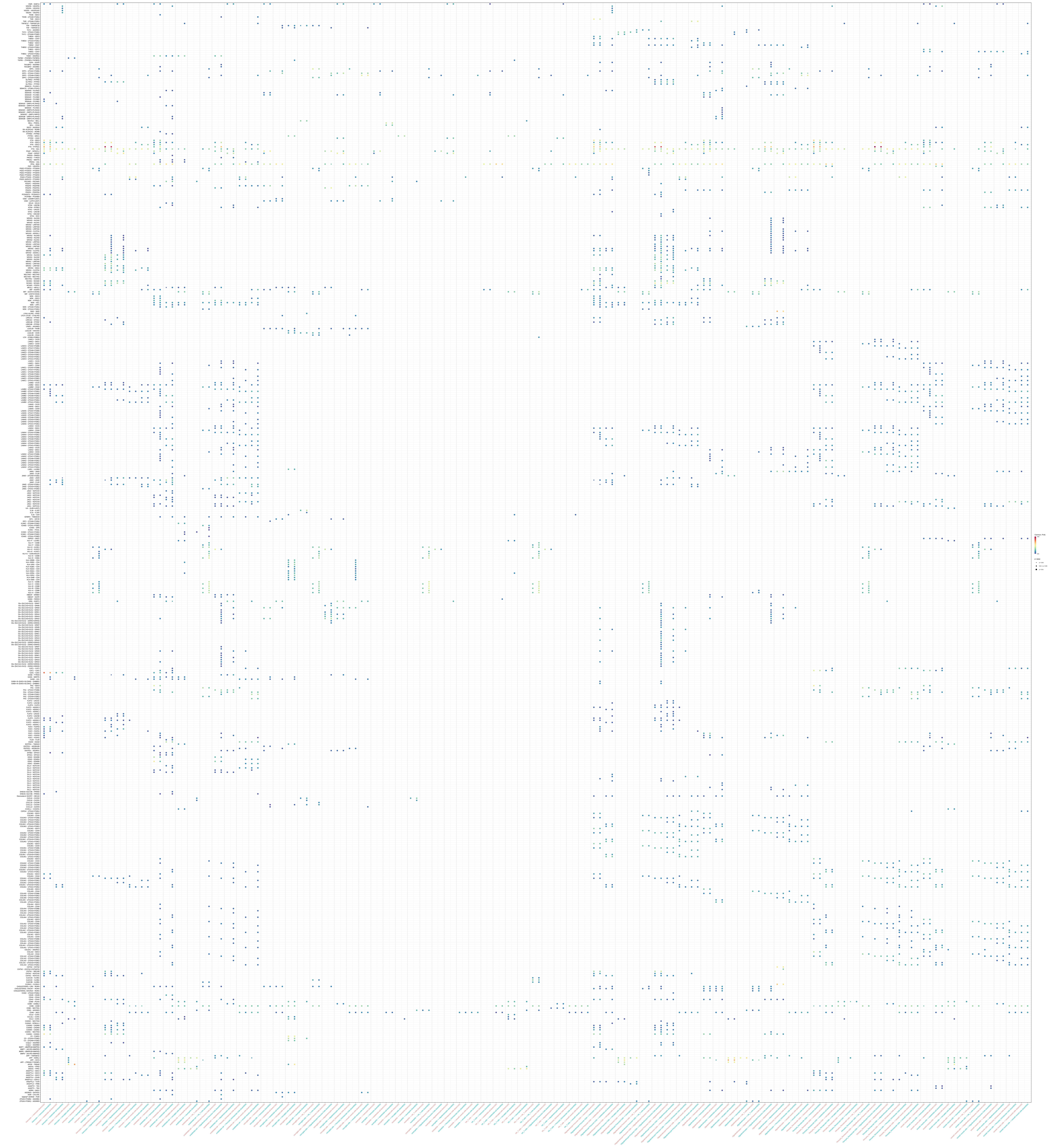

Supplement: Supplementary file 1 [file Supplementary Figure S2. Comparison_Interaction_Bubble.pdf › Supplementary Figure S2. Comparison_Interaction_Bubble.pdf]
